# Complement C5aR/LPS-induced BDNF and NGF modulation in human dental pulp stem cells

**DOI:** 10.1038/s41598-022-06110-0

**Published:** 2022-02-07

**Authors:** Muhammad Irfan, Ji Hyun Kim, Robert E. Druzinsky, Sriram Ravindran, Seung Chung

**Affiliations:** Department of Oral Biology, College of Dentistry, University of Illinois Chicago, 801 S. Paulina St, Chicago, IL 60612 USA

**Keywords:** Stem cells, Mesenchymal stem cells, Stem-cell biotechnology

## Abstract

Stem cells with the ability to differentiate into a variety of cells and secrete nerve regeneration factors have become an emerging option in nerve regeneration. Dental pulp stem cells (DPSCs) appear to be a good candidate for nerve regeneration given their accessibility, neural crest origin, and neural repair qualities. We have recently demonstrated that the complement C5a system, which is an important mediator of inflammation and tissue regeneration, is activated by lipoteichoic acid-treated pulp fibroblasts, and governs the production of brain-derived nerve growth factor (BDNF). This BDNF secretion promotes neurite outgrowth towards the injury site. Here, we extend our observation to DPSCs and compare their neurogenic ability to bone marrow-derived mesenchymal stem cells (BM-MSCs) under inflammatory stimulation. Our ELISA and immunostaining data demonstrate that blocking the C5a receptor (C5aR) reduced BDNF production in DPSCs, while treatment with C5aR agonist increased the BDNF expression, which suggests that C5aR has a positive regulatory role in the BDNF modulation of DPSCs. Inflammation induced by lipopolysaccharide (LPS) treatment potentiated this effect and is C5aR dependent. Most important, DPSCs produced significantly higher levels of C5aR-mediated BDNF compared to BM-MSCs. Taken together, our data reveal novel roles for C5aR and inflammation in modulation of BDNF and NGF in DPSCs.

## Introduction

A promising attempt to regenerate peripheral nerve involves cell-based therapies that can differentiate into neurons and/or secrete trophic factors to promote nerve regeneration^1^. Dental pulp stem cells (DPSCs) are derived from the neural crest, which also gives rise to peripheral neurons and glia^2^. Similarities to neuronal cells, such as the strong expression of neuronal marker molecules and neurotransmitter receptors, suggests that DPSCs can actively adapt to the neuronal environment^3,4^. DPSCs appear to show better neuroregeneration and protective ability than bone marrow- derived mesenchymal stem cells (BM-MSCs) in rodent ischemia models^5,6^. Furthermore, DPSCs are relatively easy to obtain through a minimally invasive procedure in adults and children. These characteristics make DPSCs an ideal stem cell source for neural regeneration. However, they have been given relatively less attention in neural regeneration compared to other stem cell types such as neural stem cells (NSCs), induced pluripotent stem cells (iPSCs), and MSCs. For example, in a PubMed search, only 34 publications were found using the search terms DPSCs and nerve, compared to 925 in a search MSC and nerve and 956 in a search for iPSC & nerve. These suggest that DPSC in neural regeneration is largely unexplored territory.

Nerve repair and regeneration require the stimulation of progenitor cells to migrate and differentiate to connect the medial and distal segments of a severed or damaged nerve. Matrix proteins are known to guide cell migration and axon growth from one segment to another and neurotrophic growth factors (nerve growth factor (NGF), brain-derived neurotrophic factor (BDNF), etc.) promote progenitor cell proliferation and differentiation^7^. While the general concepts for successful peripheral nerve regeneration have been enumerated, clinical success remains elusive. For example, the clinical administration of BDNF has been a major challenge and ineffective. The recombinant protein has a very short half-life (less than 10 min), which severely limits its effectiveness^8^. For this approach to be effective, a stable and constant BDNF production platform is crucial and stem cell engineering might fulfill this important need.

Recently, Pagella et al.^9^, have demonstrated the superiority of DPSCs over BM-MSCs in promoting trigeminal innervation by increased secretion of neurotropic factors (i.e., BDNF and NGF) and enhanced axonal growth. Previously, we have demonstrated that C5a receptor (C5aR) activity stimulates pulp fibroblast mediated BDNF secretion for neurite outgrowth. We confirmed that pulpal injury or simulated cell insult (using lipoteichoic acid (LTA) that mimics inflammatory infection by Gram-positive bacteria) cause pulp fibroblasts to express neurogenic growth factors that stimulate pulp neurite outgrowth through the C5a complement system^10–12^. Here, we investigate further the role of complement C5aR and inflammation in another important subset of regenerative cells in pulp—DPSCs—and compared their neurogenic ability to bone marrow-derived mesenchymal stem cells (BM-MSCs).

## Results

### DPSCs and BM-MSCs express BDNF and NGF

**Figure 1:**
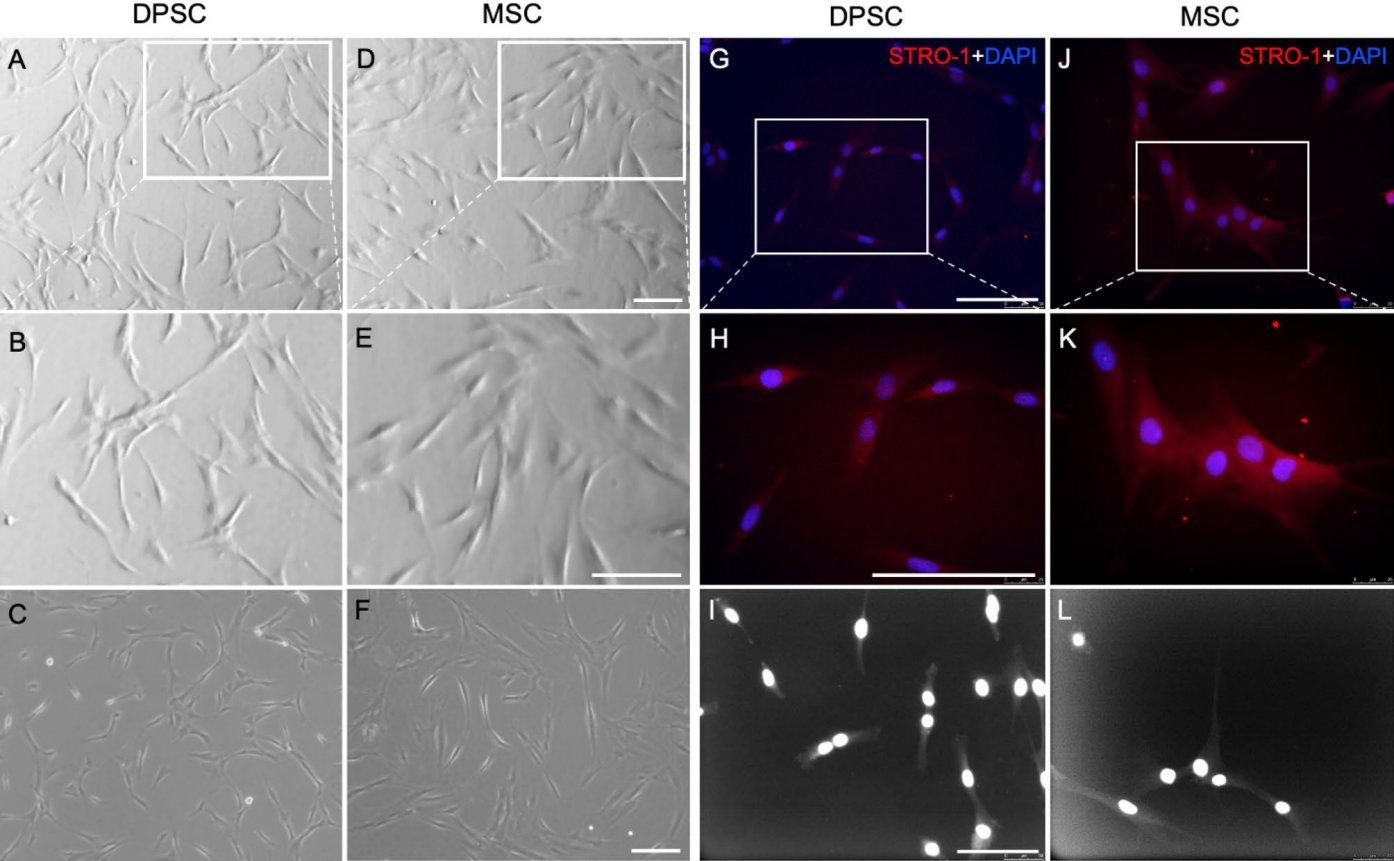
Characterization of human dental pulp stem cells and human bone marrow-derived mesenchymal stem cells. Commercially available DPSCs and BM-MSCs were acquired, and confluent cells (between 2nd and 4th passages) were cultured at 1 × 10^4^ cells concentration in a regular growth media for 3 days. (**A**–**F**) Cultured DPSCs and BM-MSCs under a light microscope at day 3. (**G**–**L**) Cells were fixed, permeabilized, and stained with anti-STRO-1 (Red) and DAPI (Blue). Immunofluorescence staining was used to visualize DPSCs and BM-MSCs marker STRO-1 sorted cells, indicated in the representative microscopic images (**G**,**H** and **J**,**K**). **I**–**L** showing fluorescence phase-contrast images of DPSCs and BM-MSCs, respectively. (**B**,**E**,**H**,**K**) are higher magnification views of the areas indicated in (**A**,**D**,**G**,**J**). All scale bars: 100 µm.

To identify the specific role of C5aR in the modulation of BDNF and NGF production in DPSCs, and to compare their neurotrophins secretion capacity to that of BM-MSCs, commercially available DPSCs and BM-MSCs were acquired and characterized by immunocytochemistry. The homogeneous populations of DPSCs and BM-MSCs were examined with the co-localization of the mesenchymal stem cell marker STRO-1. These cells have been validated in several recent publications^13–15^ and our analysis confirms over 99% purity of DPSCs and BM-MSCs. Representative images show morphological features of DPSCs and BM-MSCs (Fig. 1).

**Figure 2:**
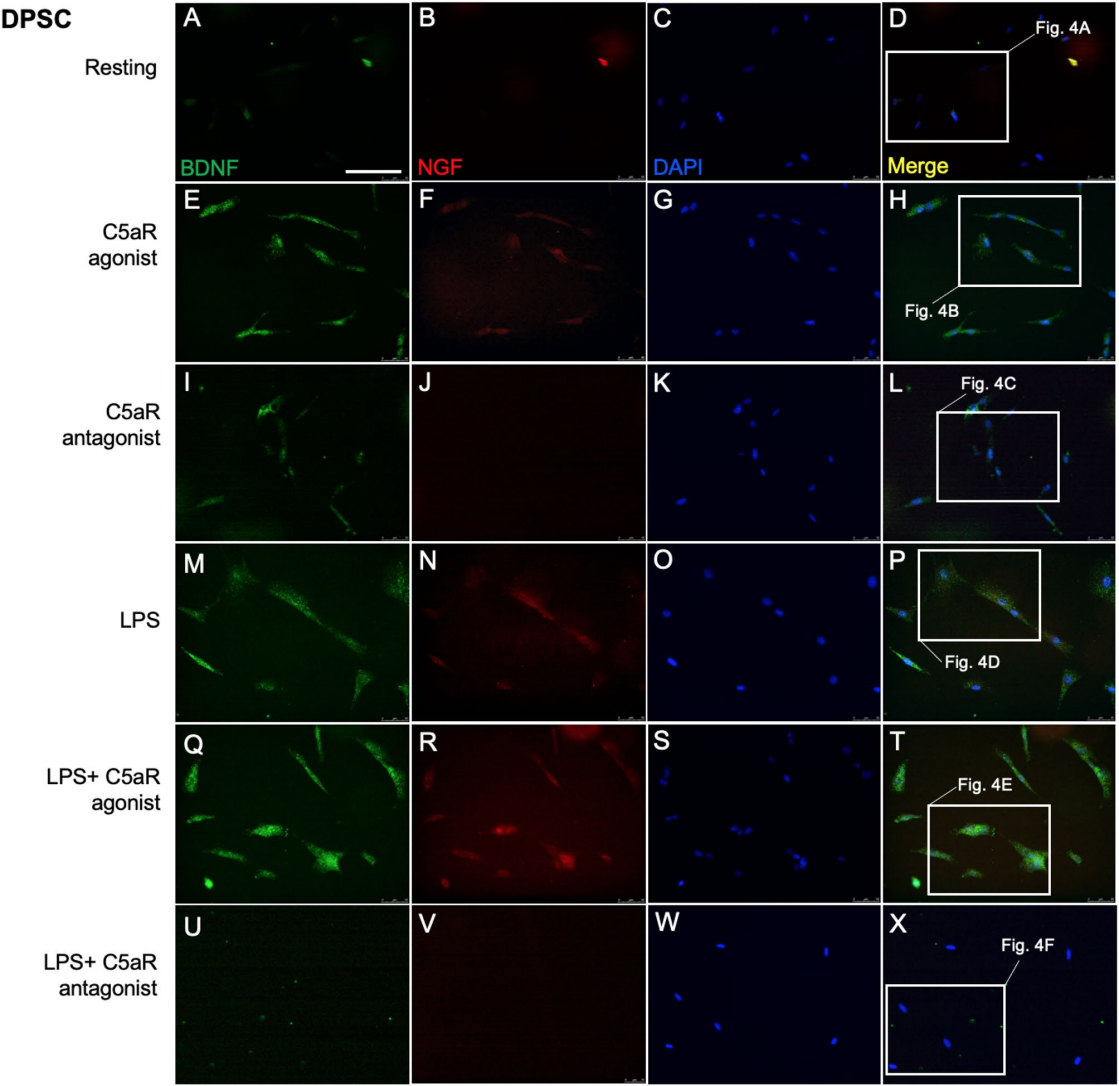
DPSCs express BDNF and NGF. Cultured cells were treated with C5aR-agonist or antagonist for 48 h and then stimulated with LPS for 1 h, and fixed and stained accordingly. Immunofluorescence double staining was used to analyze the expression of BDNF and NGF. Anti-BDNF (green) and anti-NGF (red) were found co-localized in the cytoplasm of DPSCs in C5aR-agonist treated cells after 48 h of treatment. Intense staining was observed in C5aR-agonist and LPS + C5aR-agonist treated cells. For each condition, negative controls, performed by replacement of the BDNF and NGF primary antibodies and secondary antibody used to detect BDNF was Alexa-488 (green) and NGF through Alexa-594 (red); nuclei were counterstained with DAPI (blue). Scale bar: 100 µm.

**Figure 3:**
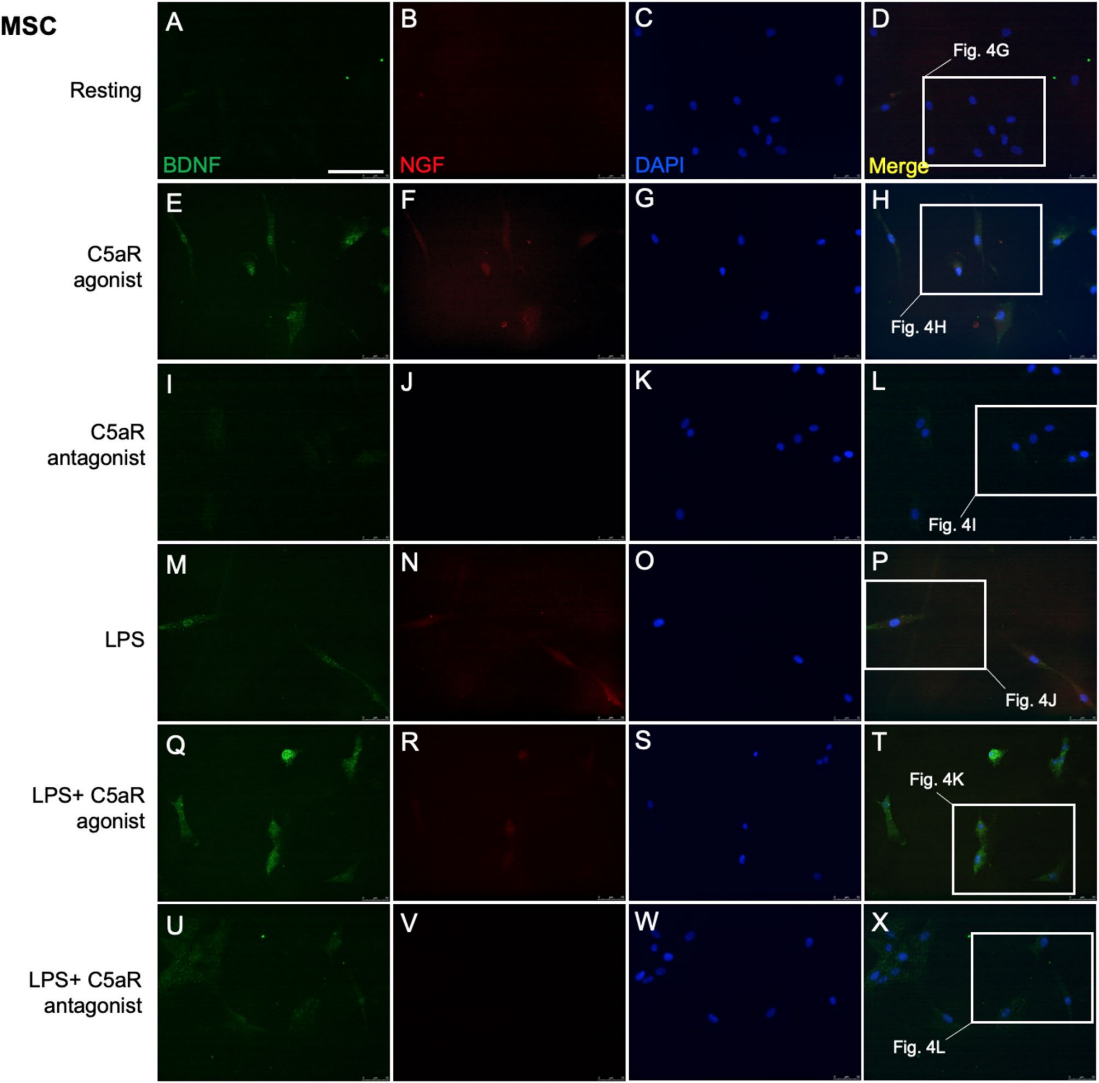
BDNF and NGF are weakly expressed in BM-MSCs. Cultured cells were treated with C5aR-agonist or antagonist for 48 h and then stimulated with LPS for 1 h, and fixed and stained accordingly. Immunofluorescence double staining was used to analyze the expression of BDNF and NGF. Anti-BDNF (green) and anti-NGF (red) were found weakly co-localized in the cytoplasm of BM-MSCs in C5aR-agonist treated cells after 48 h of treatment. Staining was observed in C5aR-agonist and LPS + C5aR-agonist treated cells. For each condition, negative controls, performed by replacement of the BDNF and NGF primary antibodies and secondary antibody used to detect BDNF was Alexa-488 (green) and NGF through Alexa-594 (red); nuclei were counterstained with DAPI (blue). Scale bar: 100 µm.

We next investigated whether DPSCs and BM-MSCs constitutively express BDNF and NGF. Double immunofluorescence staining with anti-BDNF and anti-NGF antibodies showed that both stem cells moderately express BDNF and NGF (Figs. 2A–D, 3A–D). Our ELISA further confirmed these results (Fig. 5) and these observations were consistent with previous studies^16,17^.

### C5aR modulates BDNF and NGF secretions in DPSCs and BM-MSCs

**Figure 4:**
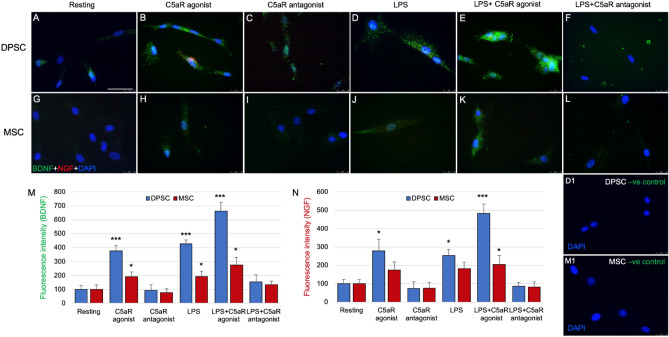
C5aR or LPS-stimulated DPSCs express more BDNF than BM-MSCs. (**A**–**L**) Magnified pictures from the respective boxes of Figs. 2 and 3 in subsequent order. Groups: Resting (**A**,**G**), C5aR agonist (**B**,**H**), C5aR antagonist (**C**,**I**), LPS (**D**,**J**), LPS + C5aR agonist (**E**,**K**), LPS + C5aR antagonist (**F**,**L**). Double fluorescence immunohistochemistry with anti-BDNF (green) and anti-NGF (red) deposit’s reaction product in punctae of both stem cells. BDNF and NGF immune intensities are significantly higher in the C5aR agonist or LPS + C5aR-agonist treatment group (**B**,**E** & **H**,**K**) compared to resting (**A**,**G**) and C5aR antagonist treatment (**C**,**I**) groups. (**M**,**N**) Bar graphs showing the fluorescence intensity of BDNF and NGF in various treatment groups normalized to untreated control of resting cells. C5aR stimulation by C5aR-agonist or co-stimulation with LPS specifically increases BDNF and NGF (E) expression in DPSCs undergoing differentiation. (D1) DPSCs negative control. (M1) BM-MSCs negative control. Values for resting control were set at 100 as a reference. *p < 0.05, **p < 0.01 and ***p < 0.001 vs untreated control of resting cells (n = 3). Scale bar: 50 μm.

Our previous study demonstrated an important role of C5aR (expressed by inflammatory pulp fibroblasts) as a positive regulator in BDNF modulation and neurite outgrowth. In the present study we examined whether the C5aR modulates BDNF and NGF secretions in DPSCs and BM-MSCs. Immunofluorescence double staining revealed expression of BDNF and NGF in the cytoplasm of DPSCs treated with C5aR-agonist (Fig. 2E–H) compared to resting (Fig. 2A–D) or C5aR antagonist treated cells (Fig. 2I–L). Co-localization of BDNF was observed in the cytoplasm of DPSCs especially around the nucleus. C5aR agonist increased the BDNF/NGF expression on DPSCs, while the treatment with C5aR antagonist decreased the BDNF/NGF expression. In detail, C5aR-agonist treatment triggered response in DPSCs to express increased BDNF (375.5 ± 37.5; *p* < 0.001; Fig. 4M) or NGF (279.2 ± 62.8; *p* < 0.05; Fig. 4N), respectively. A similar pattern was observed among C5aR-agonist treated BM-MSCs but with a weak intensity and expression i.e., BDNF (190.8 ± 34.8; *p* < 0.05; Fig. 4M) and non-significant NGF (175 ± 43.5; Fig. 4N), compared with resting control and C5aR-antagonist treated cells. Our results suggest C5aR`s positive regulatory role in the BDNF and NGF modulation on DPSCs and BM-MSCs.

### Inflammation by LPS potentiates BDNF and NGF secretions in DPSCs and BM-MSCs and it is C5aR dependent

Because we observed that C5aR expression is increased in inflammatory contexts^10–12,18^, we next examined the effect of lipopolysaccharide (LPS), which is one of the most potent single inducers of inflammation^19^, during the BDNF and NGF modulation. The LPS treatment significantly increased the BDNF and NGF expression in DPSCs and BDNF in BM-MSCs (Figs. 2M–P, 3M–P, 4D,J). Intense staining of BDNF and NGF was observed in cells treated with C5aR-agonist and LPS (Fig. 2Q–T, Fig. 4E), and C5aR-antagonist reversed the effects of LPS stimulation (Figs. 2U–X, Fig. 4L). Figure 4 shows magnified sections of DPSCs (Fig. 4A–F) and BM-MSCs (Fig. 4G–L) from Figs. 2 and 3. Briefly, LPS treatment alone triggered response in DPSCs to express increased BDNF (427.9 ± 26.8; *p* < 0.001; Fig. 4M) and NGF (252.7 ± 34.7; *p* < 0.05; Fig. 4N), respectively. A similar pattern was observed among LPS stimulated BM-MSCs but with less intensity and expression (BDNF (191.8 ± 38.4; *p* < 0.05; Fig. 4M) and non-significant NGF (181.7 ± 36.2; Fig. 4N)), compared with resting control. Notably, C5aR-agonist treatment enhanced the expression of BDNF but co-stimulation with LPS intensified the expression in DPSCs (C5aR-agonist + LPS: 662.8 ± 62.7; *p* < 0.001; Fig. 4E,M) which was comparable to the BM-MSCs with similar treatment (C5aR-agonist + LPS: 483.4 ± 50; *p* < 0.001; Fig. 4K,M). C5aR-antagonist treatment reversed the effects of LPS on BDNF expression in DPSCs (LPS + C5aR-antagonist: 153.2 ± 51.7 vs LPS: 427.9 ± 26.8; *p* < 0.001) and BM-MSCs (LPS + C5aR-antagonist: 85.6 ± 20.6 vs LPS: 191.8 ± 38.4; *p* < 0.05). The bar graph summarized the expression of BDNF and NGF, showing the clear difference among DPSCs and BM-MSCs and their ability to express these neurotrophic factors indicating that DPSCs are better at secreting neurotrophic factors than BM-MSCs. These results indicate the role of inflammation on BDNF expression in DPSCs. A similar pattern was observed among BM-MSCs but with weaker expression of BDNF or NGF (Fig. 3). Taken together, our data indicate that inflammation by LPS potentiates BDNF and NGF production in DPSCs and BM-MSCs and that this modulation is C5aR dependent.

### DPSCs secrete higher levels of BDNF than BM-MSCs

**Figure 5:**
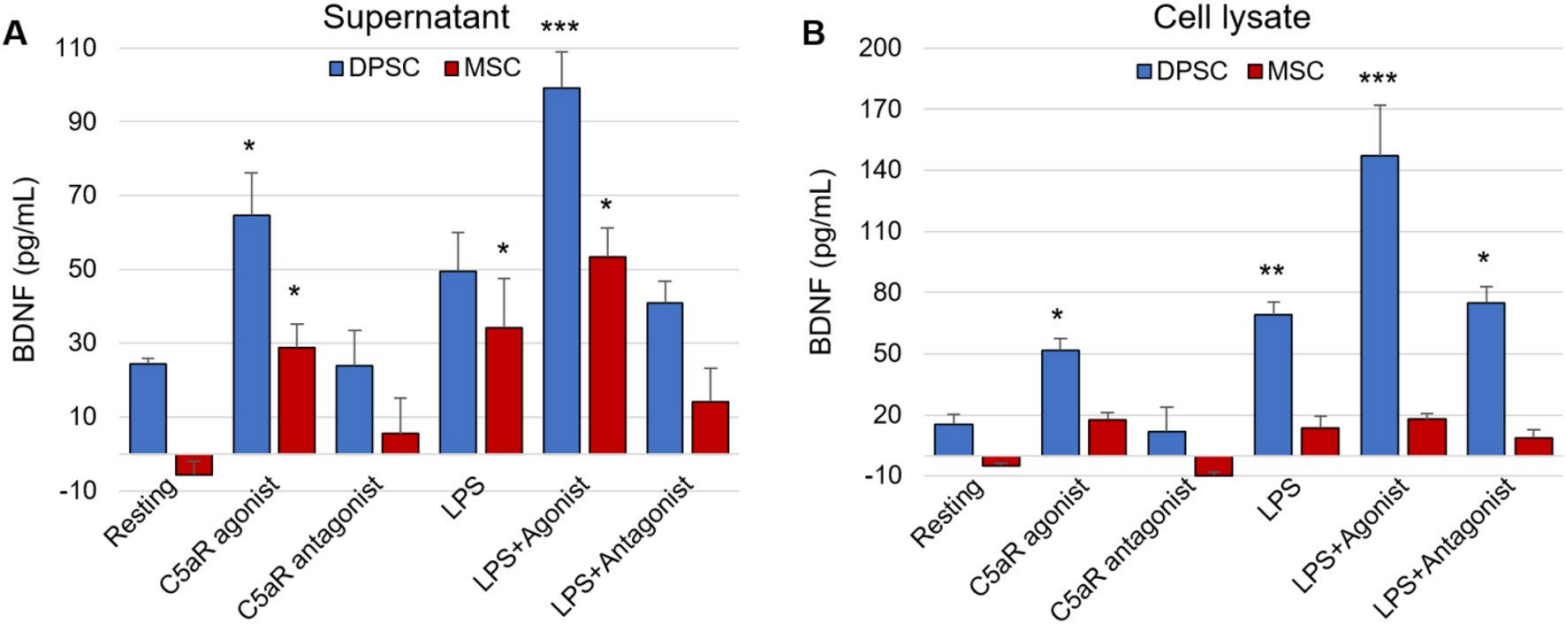
DPSCs produce higher levels of BDNF than BM-MSCs. BDNF in supernatants (**A**) and cell lysate (**B**) was measured by enzyme-linked immunosorbent assay after 48 h of treatment. For each experiment/ condition, the BDNF quantity was normalized to cell viability. Stimulation with C5aR-agonist or co-stimulation with LPS significantly increased the BDNF secretion in supernatants of DPSCs compared to BM-MSCs. BDNF secretion in the cell lysate of DPSCs was also significantly higher than BM-MSCs cell lysate. *p < 0.05, **p < 0.01 and ***p < 0.001 versus untreated control of resting cells (n = 3).

To complement the immunocytochemistry data, we performed ELISA using both supernatant and cell lysate. Figure 5A shows significant production of BDNF in supernatant in C5aR-agonist treated DPSCs (64.7 ± 11.3; *p* < 0.05) and LPS-stimulated DPSCs (49.4 ± 10.5) compared to BM-MSCs (C5aR-agonist: 28.8 ± 6.3; LPS: 34.2 ± 13.3). Co-treatment with C5aR-agonist and LPS resulted in four times the BDNF production in supernatant of DPSCs (99.1 ± 9.s7; *p* < 0.001), compared to two times the production of BDNF in BM-MSCs (53.2 ± 7.8; *p* < 0.05).

We also checked BDNF production in cell lysate and the results were similar (Fig. 5B). By themselves, C5aR-agonist or LPS treatment triggered production of BDNF (C5aR-agonist: 51.7 ± 5.7; *p* < 0.05, LPS: 68.9 ± 6.4; *p* < 0.01) while co-stimulation of C5aR-agonist and LPS potentiated its secretion (147 ± 24.9; *p* < 0.001). The C5aR-antagonist decreased BDNF production (LPS + C5aR-antagonist: 74.7 ± 8.1; *p* < 0.05) and there was almost no production of BDNF in BM-MSCs lysate (Fig. 5B). These results support the conclusion that DPSCs are better at LPS/C5aR-mediated BDNF secretion than BM-MSCs.

### Inflammation results in an increased C5aR and pp38 expression on DPSCs and BM-MSCs

**Figure 6:**
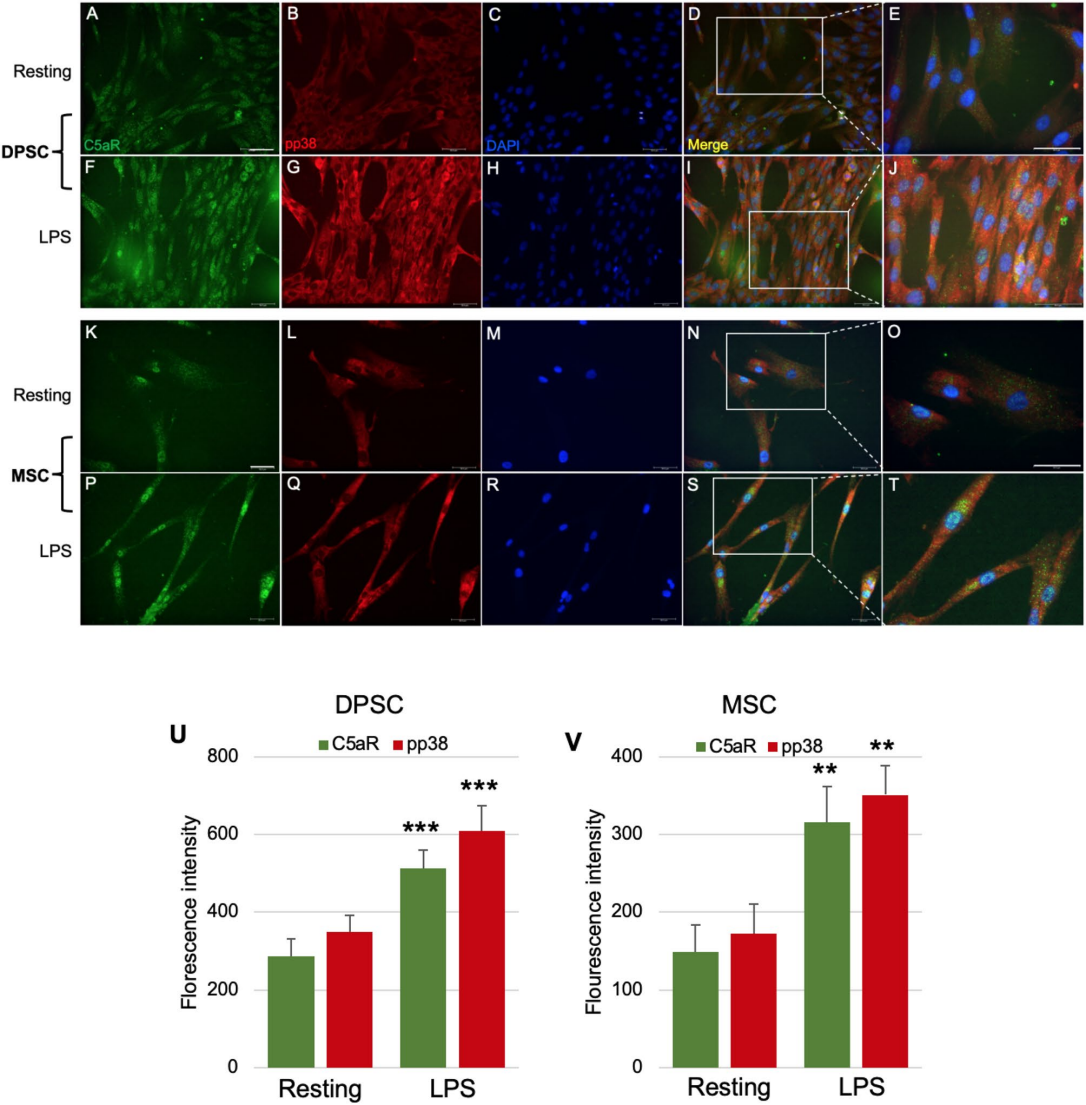
C5aR and pp38 expression in DPSCs and BM-MSCs. Cells were cultured for 3 days and then stimulated with LPS for 1 h, and fixed and stained accordingly. Immunofluorescence double staining was used to analyze the expression of C5aR and pp38. Anti-C5aR (green) and anti-pp38 (red) were found highly co-localized in the cytoplasm of DPSCs (**A**–**J**) and BM-MSCs (**K**–**T**) in LPS-stimulated cells. For each condition, negative controls, performed by replacement of the C5aR and pp38 primary antibodies and secondary antibody used to detect C5aR were Alexa-488 (green) and pp38 through Alexa-594 (red); nuclei were counterstained with DAPI (blue). (**U**,**V**) Bar graphs showing the fluorescence intensity of C5aR and pp38 in resting and LPS-stimulated DPSCs and BM-MSCs. (**E**,**J**,**O**,**T**) are higher magnification views of the areas indicated in (**D**,**I**,**N**,**S**). **p < 0.01 and ***p < 0.001 versus untreated control of resting cells (n = 3). Scale bars: 50 μm.

Complement C5a promotes proinflammatory mediators’ production in many cell types by enhancing p38 phosphorylation^20,21^ while it can also synergistically induce the production of cytokines and chemokines with LPS in various cells^22^. We also observed that p38 is required for C5aR-induced growth factor expression (unpublished observation). Thus, we have investigated the role of p38 in BDNF modulation in stem cells with respect to C5a signaling. To explore mechanistic aspects of BDNF secretion among DPSCs and BM-MSCs, we stained the LPS-stimulated DPSCs and BM-MSCs with p38 phosphorylated active form pp38. Immunofluorescence double staining revealed expression of C5aR and pp38 in the cytoplasm of DPSCs (Fig. 6A–J) and BM-MSCs (Fig. 6K–T) which homogenously co-localized. C5aR and pp38 were detected with or without LPS, and LPS treatment significantly increased the expression of C5aR and pp38 compared to controls. The merged images show an increase of p38 activation under LPS stimulation as revealed by the enhancement of yellow color, which is visible at higher magnification. No significant difference was observed in the intensity of C5aR or pp38 in DPSCs and BM-MSCs (Fig. 6U,V).

### C5aR modulation and inflammation do not affect DPSCs and BM-MSCs proliferation

**Figure 7:**
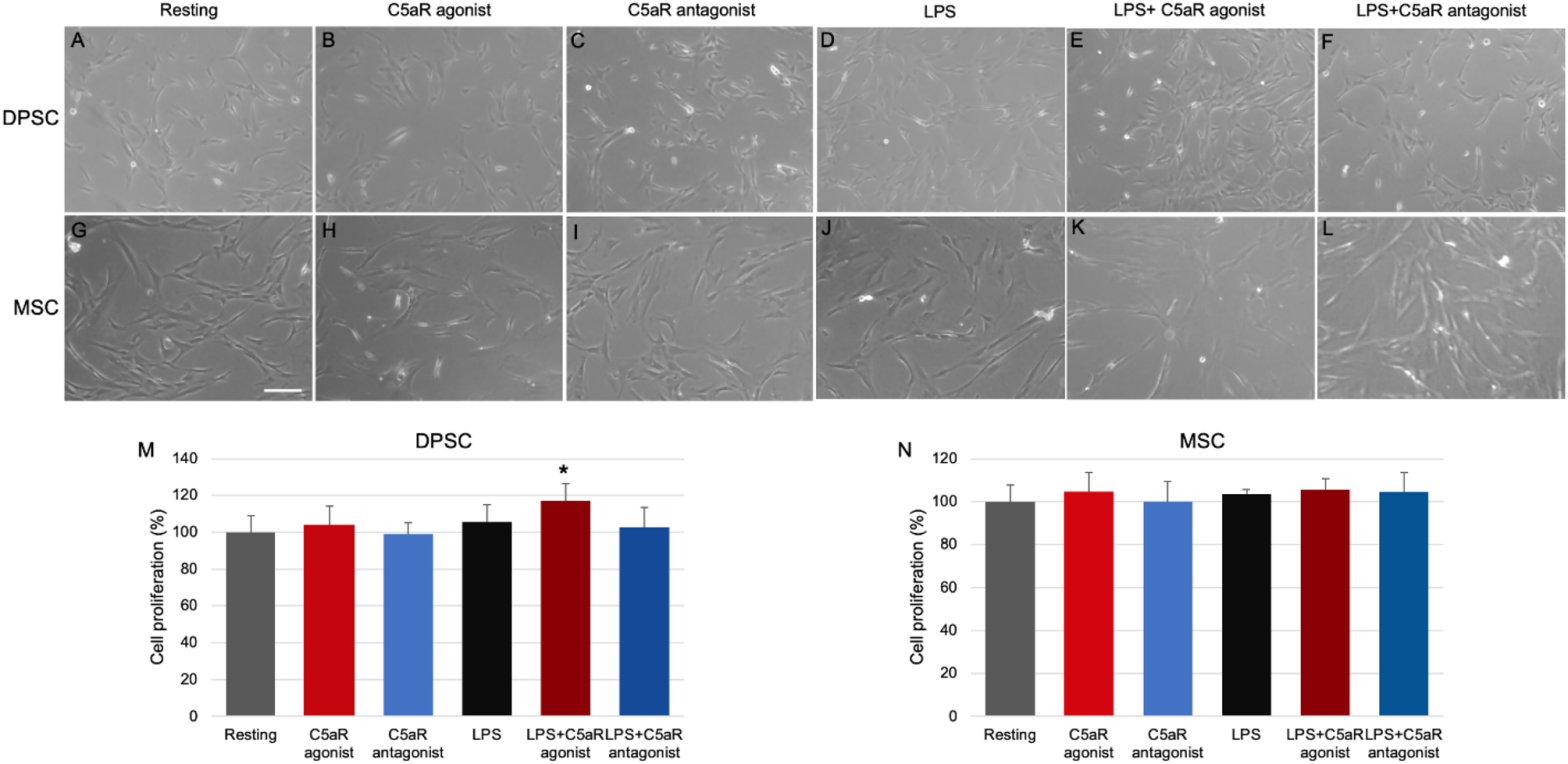
Effect of C5aR or LPS on cell proliferation of DPSCs and BM-MSCs. Cultured DPSCs (**A–F**) and MSCs (**G**–**L**) under a light microscope. Groups: Resting (**A**,**G**), C5aR agonist (**B**,**H**), C5aR antagonist (**C**,**I**), LPS (**D**,**J**), LPS + C5aR agonist (**E**,**K**), LPS + C5aR antagonist (**F**,**L**). (**M**,**N**) Bar graph showing statistical analysis—number of cells normalized as cell proliferation. Generally, C5aR modulation and LPS treatment do not affect DPSCs and BM-MSCs proliferation. *Note*: a slight increase in cell proliferation was observed in DPSCs stimulated with LPS + C5aR-agonist. Scale bar: 100 µm. *p < 0.05 versus untreated control of resting cells (n = 5).

To test whether changes in BDNF/NGF expressions are due to altered cell numbers, we next examined the effect of C5aR modulation and LPS treatment in the proliferation of DPSCs (Fig. 7A–F) and BM-MSCs (Fig. 7G–L) after 48 h of treatments. The numbers of differentiated cells were similar in all of the samples, except for a small increase in the group with the C5aR-agonist and LPS co-stimulation (*p* < 0.05; Fig. 7M). These data generally show that C5a signaling and inflammation induction do not have significant effects on the cell numbers of DPSCs (Fig. 7M) and BM-MSCs (Fig. 7N).

## Discussion

The present study demonstrates the inflammation/C5aR—mediated BDNF and NGF secretions in DPSCs. This is the first demonstration of the role of complement C5aR in BDNF/NGF production in stem cells. Our results show that C5aR controls these two neurotrophins positively. The C5aR agonist potentiated the secretion of BDNF/NGF and the C5aR antagonist reduced their production in both DPSCs and BM-MSCs. Moreover, stimulation of DPSCs with LPS significantly enhanced the production of neurotrophins and this increased production is C5aR dependent. Interestingly, our results indicate that DPSCs produced significantly higher levels of BDNF/NGF compared to BM-MSCs, which suggests that DPSCs are an emerging stem cell source that can be substituted for BM-MSCs.

Pagella et al.^9^, have recently demonstrated that DPSCs induce more extensive axonal growth than BM-MSCs, allowing formation of richer neural networks by increased secretion of neurotropic factors such as BDNF and NGF. Our previous studies have also demonstrated that pulp fibroblasts promote neurogenesis through the expression of neurotrophins and that neurotrophin expression occurs in response to injury or inflammation^10,12,18^. Our studies have successfully used lipoteichoic acid (LTA) stimulation to upregulate C5a receptors and induce pulp fibroblast neurotrophic growth factor expression (C5aR are not constitutively expressed but respond to LTA). Among the neurotrophins, we observed that BDNF is produced by dental pulp fibroblasts^10,12^. In our previous study^10^, we established that LTA-stimulated human pulp fibroblasts secrete higher levels of BDNF and enhanced axonal growth, and C5aR-antagonist limited the production of BDNF as well as decreased axonal growth. Here, we demonstrated that complement system activation induces higher levels of neurotropic secretions from DPSCs compared to BM-MSCs which could be related to the fact that enhanced BDNF production leads to increased axonal growth, as previously established^9,10^. Previous studies reported that DPSCs exhibit better neuronal stem cell properties than BM-MSCs^23^. And previous studies also demonstrated the capability of DPSCs to differentiate into neuronal cells that express neuronal markers^3,24,25^. Neurotrophins, such as BDNF and NGF, are known to have neurotrophic, neurogenic, and neuroprotective effects on the brain^26^. Recently, Sasaki et al.^27^, have shown that dental pulp-derived stem cells can differentiate into Schwann-like cells and produce neurotrophic factors including BDNF and NGF. Our results revealed the production of BDNF and NGF in C5aR-mediated DPSCs indicating the role of C5aR in the modulation of these important neurotrophins.

One of the most critical components of innate immunity and inflammation is the complement system, which can be activated via many mechanisms, such as apoptosis, necrosis, and some pathogen-associated molecular patterns^28–30^. Among the complement activation products, C5a is one of the most potent inflammatory peptides with a broad spectrum of functions. Beyond its role in immunity, the complement system participates in regeneration in liver^31^, bone^32^, and cardiac tissues^33^. Bergmann et al.^34^ explained the linkage between inflammation and dental tissue regeneration through complement activation while Liu et al.^35^ also summarized the linkage between inflammation and bone regeneration in BM-MSCs. C5a is the complement component active fragment activated by plasma proteins in response to injury. Since C5a is a powerful chemotactic factor, it is involved in one of the early steps in dentin pulp regeneration by recruitment of the immune cells and human pulp progenitor cells to the injured area^36–38^. It exerts its action by binding to the G-coupled protein receptor (GPCR) C5aR^39,40^. Gao and Yan et al.^20^, have described the involvement of complement C5a and C5aR in inflammation and sepsis. They summarized the effects of C5aR activation on various types of cells and C5aR modulation concerning endotoxins like LPS. In earlier publications we have also demonstrated that complement C5aR activation is involved in two critical steps of dentin-pulp regeneration: the pulp progenitor’s recruitment to the injured site^41,42^ and the pulp nerve sprouting beneath carious injury^10–12^.

Despite the several advantages of DPSCs in neural regeneration over other stem cell types, no investigation has systematically explored the effect of DPSCs and their derived cells in neural regeneration. As a result, our understanding of their mechanisms of action remains limited. Extracted third molars and primary teeth (both are usually considered as medical waste) are a common source of DPSCs and are much easier to obtain than other stem cells such as BM-MSCs and neural stem and progenitor cells (NSPCs). DPSCs can also be easily cryopreserved for longer periods without losing their multipotency^43^. Several studies have reported the multipotency of DPSCs including odontogenic, osteogenic, and neurogenic potential^3,44–46^, and DPSCs have been successfully used in bone tissue engineering^47,48^. BM-MSCs are multipotent stem cells characterized by self-renewal and multilineage differentiation. DPSCs display MSC-like characteristics but with easy accessibility, noninvasive isolation, limited ethical concerns, and high proliferation capacity. DPSCs are thought to be promising stem cell sources for clinical use^49^. Yamada et al.^49^, has summarized the regenerative capabilities of DPSCs in various systemic diseases, especially with regard to neural regenerative capacity compared to BM-MSCs, and concluded that DPSCs have comparatively much greater therapeutic potential. Recently, Xiao et al.^50^, have proved that DPSCs can stimulate the proliferation of neuronal cells (mainly neurons) both along the edge and inside of hippocampal slices and that they express neurotrophic factor BDNF in the Golgi complex. Another study has reported that DPSCs have higher regeneration potential than BM-MSCs^51^. Mead et al.^17^, and Pagella et al.^9^, both concluded that DPSCs produce more neurotrophic factors than BM-MSCs. And several other in vitro and in vivo studies^52–55^ reported that increased production of neurotropic factors (especially BDNF) in response to injury or inflammatory response, may enhance axonal growth and peripheral nerve regeneration. Similarly, another study^56^ revealed the in vivo performance of stem cells during tissue regeneration and demonstrated that transplantation of differentiating stem cells which secrete neurotropic factors, especially BDNF, accelerated functional nerve recovery through paracrine signaling. Our results are consistent with the above-mentioned studies, and we found that triggering C5aR or inducing a small inflammatory response in DPSCs could potentiate the secretion of important neurotrophins. In an unpublished observation, we confirmed that BDNF-overexpressing DPSCs could enhance inferior alveolar nerve regeneration 3 weeks after denervation in the mouse model.

In conclusion, our results demonstrate that DPSCs are promising alternative sources of multipotent BM-MSCs and could be a better therapeutic choice for stem cell-mediated neural regeneration.

## Material and methods

### Chemicals and reagents

Commercially available human DPSCs and human BM-MSCs (Catalogue # PT-5025 & PT-2501, respectively) were purchased from Pharma & Biotech (Lonza group Ltd, USA). C5aR antagonist—W54011 was acquired from Calbiochem (San Diego, CA, USA) and C5aR agonist from Anaspec (Fremont, CA, USA). MEM-alpha, PBS, fetal bovine serum, L-glutamine, and Antibiotic–Antimycotic were procured from Gibco Fisher Scientific (Waltham, MA, USA). Poly-D-Lysine coated (BioCoat, 12 mm) round German glass coverslips slips were purchased from Corning Fisher Scientific (Waltham, MA, USA). RIPA buffer was from Cell Signaling Technology (Danvers, MA, USA) and BDNF ELISA kit from R&D System (Minneapolis, MN, USA). Various antibodies were procured: anti-C5a receptor from Proteintech (ST. Louis, MO, USA), rabbit anti-BDNF from NovusBio (Centennial, CO, USA), mouse anti-NGF from BioLegends (San Diego, CA, USA), mouse anti-STRO-1, and mouse anti-pp38 from Santa Cruz (Dallas, Texas, USA).

### Cell culture

Human DPSCs were purchased from Pharma & Biotech, which were guaranteed through 10 population doublings, to express CD105, CD166, CD29, CD90, and CD73, and to not express CD34, CD45, and CD133.

Bone marrow-derived human Mesenchymal Stem Cells (BM-MSCs) were also purchased from Pharma and Biotech, which were guaranteed to have at least 0.75 million viable cells per vial, differentiate down the adipogenic, chondrogenic, and osteogenic lineages when cultured in the recommended differentiation medium, to express CD29, CD44, CD73, CD90, CD105, and CD166 and to not express CD14, CD19, CD34, and CD45 or HLA-DR.

We further evaluated DPSCs and BM-MSCs in cultures with STRO-1, a stem cell marker. Our analysis confirms that over 99% of cells (N = 5) used for our study were DPSCs and BM-MSCs. DPSCs and BM-MSCs were cultured at 37˚C and 5% CO_2_ treated with C5aR antagonist—W54011 (10 nM) or C5aR agonist (20 nM) for 48 h in regular growth media (α MEM containing 10% fetal bovine serum (FBS), 1% L-glutamine and antimycotic/antibiotic) and/or then stimulated with LPS (1 µg/mL) for 1 h. The experiments were conducted with different sets of DPSCs (between 2^nd^ and 4^th^ passages) 3 times.

### Immunofluorescence staining

DPSCs and BM-MSCs were fixed and permeabilized as previously described^10,12^. Subsequently, cells were incubated overnight with rabbit anti-C5a receptor (1:1000), mouse anti-p-p38 (1:1000), mouse anti-STRO-1 (1:1000), rabbit anti-BDNF (1:1000), mouse anti-NGF (1:1000) or their respective control/isotypes. Later, the cells were treated with secondary antibody for 3 h with a mix of Alexa Fluor-594 anti-mouse IgG, Alexa Fluor-488 anti-rabbit IgG (1 μg/mL), and/or DAPI (2 μg/mL). The coverslips were mounted, and images were taken using a Zeiss Axiovert microscope. Fluorescence density was quantified using ImageJ 1.49v software and values were analyzed for statistical significance by SAS 9.4.

### BDNF ELISA

Supernatants or cell lysates from DPSCs and BM-MSCs cultures, incubated with various above-mentioned treatments, were collected from cultures after 48 h and assayed using BDNF ELISA kit according to manufacturer’s protocol (R&D Systems). Briefly, a standard curve was constructed using standards and test samples in duplicate at increasing concentrations and values were normalized accordingly.

### Data analysis

The statistical analyses were performed on at least 3 independent experiments with duplicates or triplicates, and statistical significance was determined using one-way analysis of variance (ANOVA) followed by post-hoc Dunnett’s test (SAS 9.4) to compare the different treatments and their respective controls (*p* value of 0.05 or less was considered statistically significant). For quantification of immunofluorescence staining intensity, ImageJ 1.49v software was used. Fixed areas of 1 mm × 1 mm or 2 mm × 2 mm were selected to analyze the number or fluorescence intensity of differentiated cells.
